# Supplementary material for: Semiparametric methods for estimation of a nonlinear exposure‐outcome relationship using instrumental variables with application to Mendelian randomization
Source: Genet Epidemiol. 2017 Mar 20;41(4):341–52. doi: 10.1002/gepi.22041 (PMC5400068; doi:10.1002/gepi.22041)
Supplement: Supplementary file 1 — Table S1 Simulation results for fractional polynomials of degree 1. Table S2 Simulation results for fractional polynomials of degree 2 with β1 = 1 and β2 = 2. Table S3 Simulations to assess the impact of violations of the assumptions for the fractional polynomial method. Table S4 Simulations to assess the impact of violations of the assumptions for the piecewise linear method assuming a threshold effect with β = 0.5 Table S5 Additional simulations to assess the impact of varying the effect of the IV on the exposure for the fractional polynomial method. Table S6 Additional simulations to assess the impact of varying the effect of the IV on the exposure for the piecewise linear method assuming a threshold effect with β = 0:5. Figure S1 Causal effects of body mass index (BMI) on blood pressure (systolic blood pressure, SBP; diastolic blood pressure, DBP) in individuals with no history of hypertension using the fractional polynomial and piecewise linear methods on data from UK Biobank. The red point represents the reference point of BMI of 25 kg/m2. Grey lines represent 95 fractional polynomial method used 100 strata, whereas the piecewise linear method used 10 strata. [file GEPI-41-341-s001.pdf]

Semiparametric methods for estimation of a non-linear exposure–outcome relationship using instrumental variables with application to Mendelian randomization: Supplementary Data

James R Staley[1] & Stephen Burgess[1]

[1]Cardiovascular Epidemiology Unit, Department of Public Health and Primary Care, University of Cambridge, United Kingdom.

Correspondence:

Dr Stephen Burgess

Department of Public Health & Primary Care

Strangeways Research Laboratory

Wort's Causeway

Cambridge

CB1 8RN

UK

Email: sb452@medschl.cam.ac.uk

Telephone: +44 (0) 1223 748651

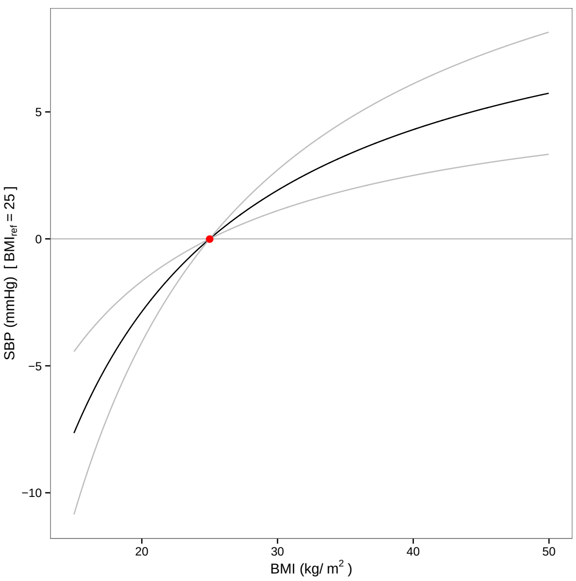

(a) SBP (Fractional polynomial model)

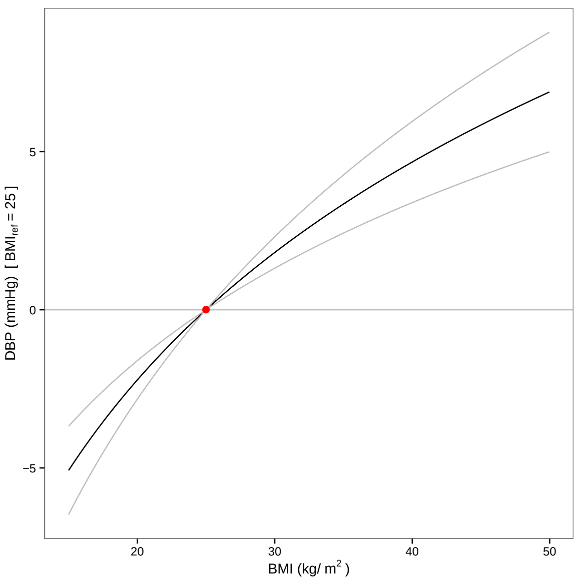

(b) DBP (Fractional polynomial model)

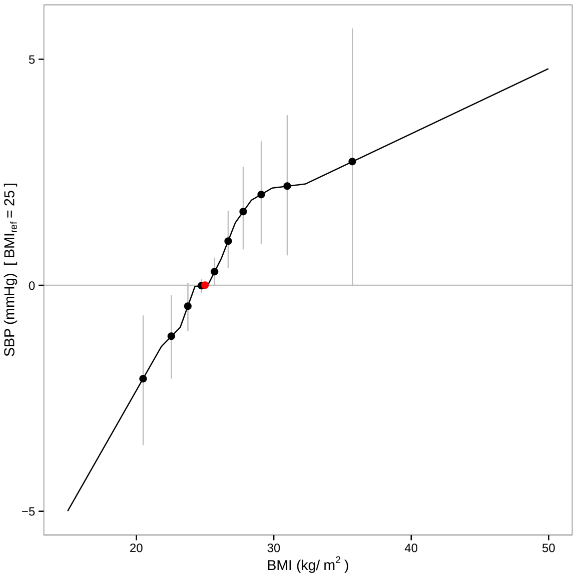

(c) SBP (Piecewise linear model)

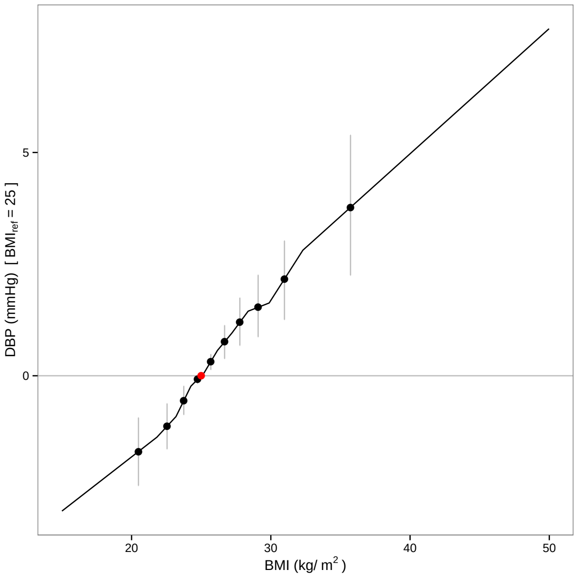

(d) DBP (Piecewise linear model)

Figure S1 Causal effects of body mass index (BMI) on blood pressure (systolic blood pressure, SBP; diastolic blood pressure, DBP) in individuals with no history of hypertension using the fractional polynomial and piecewise linear methods on data from UK Biobank. The red point represents the reference point of BMI of 25 kg/m^2. Grey lines represent 95%CIs. The fractional polynomial method used 100 strata, whereas the piecewise linear method used 10 strata.

Table S1: Simulation results for fractional polynomials of degree 1.

		Fitting correct FP		Fitting all FPs			
		Mean (SD) [Mean SE]	Coverage	Powers		Heuristic	
p	β	$\hat{\beta}$	$\hat{\beta}$	Correct	Set	Correct FP	Best FP
-2	0	0.030 (0.953) [0.933]	0.946	-	-	-	-
-2	1	1.006 (0.924) [0.929]	0.960	0.434	0.952	1.14 (0.87)	1.21 (0.85)
-2	2	2.012 (0.956) [0.933]	0.928	0.496	0.942	1.17 (0.92)	1.29 (0.92)
-1	0	0.046 (0.700) [0.646]	0.932	-	-	-	-
-1	1	0.936 (0.635) [0.643]	0.956	0.070	0.950	1.03 (0.80)	1.13 (0.84)
-1	2	1.921 (0.654) [0.641]	0.946	0.142	0.956	1.06 (0.81)	1.21 (0.82)
-0.5	0	-0.013 (0.774) [0.742]	0.922	-	-	-	-
-0.5	1	0.981 (0.724) [0.743]	0.946	0.050	0.946	0.95 (0.75)	1.07 (0.79)
-0.5	2	1.959 (0.752) [0.740]	0.938	0.088	0.912	0.99 (0.78)	1.17 (0.83)
0	0	-0.009 (0.221) [0.211]	0.934	-	-	-	-
0	1	0.979 (0.215) [0.210]	0.944	0.172	0.918	0.95 (0.74)	1.20 (0.79)
0	2	1.976 (0.210) [0.212]	0.954	0.386	0.910	0.94 (0.72)	1.22 (0.76)
0.5	0	0.000 (0.246) [0.233]	0.930	-	-	-	-
0.5	1	1.003 (0.246) [0.234]	0.936	0.194	0.892	0.93 (0.70)	1.19 (0.75)
0.5	2	1.987 (0.239) [0.235]	0.932	0.340	0.904	0.91 (0.68)	1.21 (0.79)
1	0	0.003 (0.064) [0.064]	0.938	-	-	-	-
1	1	0.997 (0.066) [0.065]	0.944	0.748	0.938	0.88 (0.64)	1.12 (0.79)
1	2	1.997 (0.071) [0.068]	0.938	0.912	0.958	0.92 (0.70)	1.08 (0.91)
2	0	0.000 (0.009) [0.008]	0.942	-	-	-	-
2	1	1.015 (0.012) [0.012]	0.756	1.000	1.000	1.66 (1.07)	1.66 (1.07)
2	2	2.031 (0.015) [0.015]	0.436	1.000	1.000	3.16 (1.49)	3.16 (1.49)
3	0	0.000 (0.001) [0.001]	0.942	-	-	-	-
3	1	1.034 (0.006) [0.005]	0.000	1.000	1.000	17.75 (3.10)	17.75 (3.10)
3	2	2.068 (0.011) [0.009]	0.000	1.000	1.000	35.74 (5.64)	35.74 (5.64)

p is the power, and β is the effect parameter. Coverage refers to the number of replications where the true β was contained within the corresponding 95%CI. The power was correctly chosen (Correct) if the best-fitting fractional polynomial was also the correct fractional polynomial, whilst the correct model was within the set of powers that fit the data equally as well as the best-fitting fractional polynomial (Set) if the difference between twice the log-likelihood for the correct model and the best-fitting model was less than the 90th percentile of the relevant χ^2 distribution. The heuristic statistic (mean (SD) across simulations) is the sum of the absolute values of the predicted value of the outcome minus the correct value of the outcome at the mean value of the exposure in deciles of the IV-free distribution. The heuristic statistic is calculated both for the correct fractional polynomial (Correct FP), and for the best-fitted fractional polynomial (Best FP). SD, standard deviation; SE, standard error; FP, fractional polynomial; IV, instrumental variable; CI, confidence interval.

Table S2: Simulation results for fractional polynomials of degree 2 with $\beta_1 = 1$ and $\beta_2 = 2$.

		Fitting correct FP				Fitting all FPs			
		Mean (SD) [Mean SE]		Coverage		Powers		Heuristic	
p_1	p_2	$\hat{\beta}_1$	$\hat{\beta}_2$	$\hat{\beta}_1$	$\hat{\beta}_2$	Correct	Set	Correct FP	Best FP
-2	-2	0.856 (3.756) [3.606]	2.178 (6.653) [6.502]	0.926	0.942	0.272	0.988	1.55 (1.08)	1.48 (1.02)
-2	-1	0.433 (5.995) [5.994]	2.281 (4.170) [4.160]	0.950	0.950	0.012	0.974	1.41 (0.91)	1.43 (0.89)
-2	-0.5	1.305 (4.041) [3.833]	1.784 (3.173) [3.070]	0.934	0.944	0.010	0.986	1.45 (0.93)	1.44 (0.94)
-2	0	0.812 (2.786) [2.805]	1.955 (0.624) [0.636]	0.946	0.950	0.026	0.958	1.34 (0.83)	1.48 (0.91)
-2	0.5	0.954 (2.142) [2.187]	1.999 (0.552) [0.552]	0.958	0.954	0.036	0.964	1.28 (0.79)	1.44 (0.93)
-2	1	1.102 (1.887) [1.974]	2.003 (0.135) [0.140]	0.948	0.948	0.072	0.964	1.40 (0.88)	1.67 (1.07)
-2	2	0.310 (2.830) [2.514]	2.025 (0.034) [0.030]	0.908	0.828	0.314	0.964	3.91 (1.73)	5.01 (2.70)
-2	3	-6.114 (8.221) [6.580]	2.051 (0.023) [0.018]	0.812	0.280	0.440	0.980	37.67 (5.59)	34.6 (6.79)
-1	-1	1.004 (0.713) [0.718]	2.067 (3.855) [3.776]	0.946	0.946	0.008	0.980	1.31 (0.87)	1.37 (0.95)
-1	-0.5	0.525 (7.081) [7.247]	2.551 (8.175) [8.363]	0.952	0.948	0.002	0.962	1.31 (0.87)	1.38 (0.91)
-1	0	1.399 (3.551) [3.505]	2.109 (1.184) [1.146]	0.944	0.938	0.006	0.968	1.30 (0.78)	1.47 (0.91)
-1	0.5	0.815 (2.166) [2.265]	1.929 (0.784) [0.824]	0.954	0.958	0.006	0.964	1.29 (0.83)	1.49 (0.99)
-1	1	0.871 (1.915) [1.887]	1.985 (0.193) [0.192]	0.942	0.938	0.004	0.962	1.33 (0.79)	1.70 (1.05)
-1	2	0.293 (2.488) [2.204]	2.020 (0.043) [0.037]	0.900	0.902	0.026	0.956	3.74 (1.54)	4.73 (2.61)
-1	3	-3.729 (6.988) [5.521]	2.051 (0.029) [0.022]	0.820	0.432	0.026	0.966	36.58 (5.29)	34.62 (6.74)
-0.5	-0.5	1.021 (3.551) [3.588]	1.959 (3.903) [3.974]	0.938	0.946	0.018	0.990	1.28 (0.81)	1.37 (0.86)
-0.5	0	1.113 (7.621) [7.615]	2.008 (2.159) [2.158]	0.956	0.952	0.002	0.982	1.25 (0.84)	1.44 (0.99)
-0.5	0.5	1.296 (3.715) [3.688]	2.093 (1.162) [1.162]	0.944	0.946	0.002	0.968	1.20 (0.79)	1.5 (1.05)
-0.5	1	0.752 (2.843) [2.787]	1.979 (0.246) [0.246]	0.944	0.946	0.002	0.938	1.30 (0.79)	1.76 (1.08)
-0.5	2	0.314 (3.249) [3.012]	2.022 (0.049) [0.044]	0.922	0.896	0.012	0.974	3.66 (1.52)	4.51 (2.33)
-0.5	3	-5.579 (8.982) [7.322]	2.049 (0.031) [0.025]	0.836	0.500	0.022	0.954	36.60 (5.48)	35.21 (7.03)
0	0	0.891 (1.287) [1.336]	2.036 (0.553) [0.576]	0.952	0.956	0.008	0.966	1.23 (0.78)	1.64 (1.07)
0	0.5	0.996 (2.106) [2.033]	1.977 (2.346) [2.262]	0.938	0.934	0.004	0.964	1.21 (0.72)	1.53 (0.95)
0	1	1.004 (1.149) [1.172]	1.996 (0.353) [0.362]	0.940	0.940	0.012	0.948	1.22 (0.77)	1.69 (1.10)
0	2	1.303 (1.248) [1.087]	2.017 (0.064) [0.055]	0.898	0.902	0.026	0.954	3.72 (1.46)	4.88 (2.60)
0	3	3.223 (2.986) [2.469]	2.044 (0.036) [0.029]	0.840	0.610	0.026	0.968	35.52 (5.56)	34.02 (7.52)
0.5	0.5	1.031 (4.390) [3.986]	1.979 (1.377) [1.252]	0.912	0.918	0.006	0.952	1.3 (0.79)	1.72 (1.09)
0.5	1	1.025 (2.603) [2.557]	1.996 (0.716) [0.705]	0.938	0.940	0.010	0.954	1.27 (0.74)	1.76 (1.11)
0.5	2	1.477 (1.784) [1.633]	2.011 (0.079) [0.072]	0.936	0.934	0.024	0.970	3.64 (1.53)	4.9 (2.66)
0.5	3	3.956 (3.832) [3.399]	2.040 (0.039) [0.034]	0.864	0.774	0.016	0.964	34.85 (5.68)	34.91 (7.20)
1	1	1.071 (0.990) [0.959]	1.979 (0.452) [0.438]	0.942	0.936	0.086	0.966	1.47 (0.83)	2.14 (1.33)
1	2	1.231 (0.776) [0.703]	1.995 (0.121) [0.110]	0.912	0.908	0.030	0.960	3.64 (1.42)	4.88 (2.58)
1	3	2.180 (1.425) [1.224]	2.028 (0.051) [0.042]	0.826	0.846	0.038	0.968	34.2 (5.94)	34.48 (7.81)
2	2	1.233 (0.362) [0.306]	1.920 (0.219) [0.184]	0.858	0.900	0.246	0.944	10.44 (2.47)	12.35 (6.31)
2	3	1.389 (0.504) [0.419]	1.990 (0.108) [0.089]	0.846	0.918	0.136	0.948	34.82 (7.11)	39.65 (11.21)
3	3	1.377 (0.295) [0.233]	1.864 (0.202) [0.157]	0.660	0.842	0.704	0.966	84.13 (17.71)	74.39 (18.89)

β_1 and β_2 are the effect parameters, and p_1 and p_2 are the powers. Coverage refers to the number of replications where the true β was contained within the corresponding 95%CI. The powers were correctly chosen (Correct) if the best-fitting fractional polynomial was also the correct fractional polynomial, whilst the correct model was within the set of powers that fit the data equally as well as the best-fitting fractional polynomial (Set) if the difference between twice the log-likelihood for the correct model and the best-fitting model was less than the 90th percentile of the relevant χ^2 distribution. The heuristic statistic (mean (SD) across simulations) is the sum of the absolute values of the predicted value of the outcome minus the correct value of the outcome at the mean value of the exposure in deciles of the IV-free distribution. The heuristic statistic is calculated both for the correct fractional polynomial (Correct FP), and for the best-fitted fractional polynomial (Best FP). SD, standard deviation; SE, standard error; FP, fractional polynomia; IV, instrumental variable; CI, confidence interval.

Table S3: Simulations to assess the impact of violations of the assumptions for the fractional polynomial method.

			Fitting correct FP		Power of test		
Simulation	Model	β	Mean (SD) [Mean SE]	Coverage	Quad	Q	FP
IV–exposure	Linear	0	0.003 (0.064) [0.064]	0.940	0.048	0.078	0.020
	Linear	1	1.001 (0.063) [0.065]	0.952	0.036	0.046	0.022
	Logarithm	2	1.967 (0.226) [0.211]	0.926	0.550	0.274	0.582
	Square root	2	1.974 (0.242) [0.235]	0.934	0.174	0.094	0.176
	Quadratic	0.1	0.101 (0.009) [0.009]	0.954	0.612	0.318	0.586
Exposure–outcome	Linear	0	-0.001 (0.079) [0.078]	0.938	0.050	0.058	0.006
	Linear	1	1.003 (0.080) [0.078]	0.944	0.044	0.068	0.030
	Logarithm	2	1.977 (0.220) [0.216]	0.938	0.456	0.230	0.496
	Square root	2	1.980 (0.248) [0.250]	0.946	0.144	0.080	0.140
	Quadratic	0.1	0.101 (0.026) [0.024]	0.940	0.114	0.098	0.092
Both	Linear	0	0.009 (0.081) [0.077]	0.936	0.062	0.060	0.012
	Linear	1	0.991 (0.080) [0.078]	0.930	0.056	0.070	0.042
	Logarithm	2	1.972 (0.218) [0.216]	0.942	0.490	0.250	0.516
	Square root	2	2.005 (0.250) [0.250]	0.960	0.134	0.092	0.138
	Quadratic	0.1	0.103 (0.024) [0.024]	0.956	0.128	0.100	0.116
Both ($\rho = 0.2$)	Linear	0	0.015 (0.082) [0.077]	0.938	0.114	0.086	0.022
	Linear	1	1.004 (0.076) [0.078]	0.956	0.114	0.064	0.084
	Logarithm	2	1.957 (0.208) [0.218]	0.950	0.352	0.162	0.382
	Square root	2	1.997 (0.258) [0.248]	0.938	0.078	0.064	0.088
	Quadratic	0.1	0.100 (0.026) [0.024]	0.924	0.668	0.408	0.576

IV–exposure refers to the simulation setting where the IV–exposure association was allowed to vary across individuals. Exposure–outcome refers to the simulation setting where the exposure–outcome association was allowed to vary across individuals. Both refers to the simulation setting where both the IV–exposure and exposure–outcome associations were allowed to vary across individuals. Both ($\rho = 0.2$) refers to the simulation setting where both the IV–exposure and exposure–outcome associations were allowed to vary across individuals with correlation of 0.2. β is the effect parameter. Coverage refers to the number of replications where the true β was contained within the corresponding 95%CI. SD, standard deviation; SE, standard error; Quad, quadratic test for assessing non-linearity; Q, Cochran-Q test; FP; fractional polynomial test; IV, instrumental variable; CI, confidence interval.

Table S4: Simulations to assess the impact of violations of the assumptions for the piecewise linear method assuming a threshold effect with $\beta = 0.5$

(a) Overall results

	Power of test		
Varying	Quad	Q	FP
IV–exposure	0.848	0.706	0.772
Exposure–outcome	0.826	0.638	0.716
Both	0.774	0.644	0.708
Both ($\rho = 0.2$)	0.858	0.712	0.792

(b) Quantile results

		Decile of the IV-free exposure distribution									
Simulation	Parameter	1	2	3	4	5	6	7	8	9	10
IV–exposure	Correct	0.000	0.000	0.000	0.000	0.000	0.000	0.057	0.227	0.488	1.182
	Mean	0.023	0.039	0.044	0.039	0.033	0.045	0.115	0.275	0.543	1.244
	Coverage	0.934	0.928	0.934	0.938	0.934	0.918	0.908	0.932	0.934	0.926
Exposure–outcome	Correct	0.000	0.000	0.000	0.000	0.000	0.000	0.054	0.224	0.486	1.179
	Mean	0.005	0.007	0.005	0.004	0.004	0.015	0.080	0.235	0.498	1.182
	Coverage	0.936	0.924	0.924	0.932	0.930	0.926	0.932	0.948	0.952	0.950
Both	Correct	0.000	0.000	0.000	0.000	0.000	0.000	0.057	0.227	0.488	1.182
	Mean	0.025	0.041	0.045	0.042	0.038	0.048	0.116	0.270	0.530	1.222
	Coverage	0.936	0.926	0.938	0.936	0.928	0.932	0.922	0.924	0.924	0.942
Both ($\rho = 0.2$)	Correct	0.000	0.000	0.000	0.000	0.000	0.000	0.057	0.227	0.488	1.182
	Mean	0.028	0.045	0.049	0.043	0.036	0.046	0.115	0.275	0.542	1.286
	Coverage	0.934	0.936	0.926	0.932	0.950	0.942	0.940	0.940	0.942	0.936

IV–exposure refers to the simulation setting where the IV–exposure association was allowed to vary across individuals. Exposure–outcome refers to the simulation setting where the exposure–outcome association was allowed to vary across individuals. Both refers to the simulation setting where both the IV–exposure and exposure–outcome associations were allowed to vary across individuals. Both ($\rho = 0.2$) refers to the simulation setting where both the IV–exposure and exposure–outcome associations were allowed to vary across individuals with correlation of 0.2. Coverage refers to the number of replications where the true mean value of the outcome for that decile was contained within the corresponding 95% prediction interval. Quad, quadratic test for assessing non-linearity; Q, Cochran-Q test; FP; fractional polynomial test; IV, instrumental variable.

Table S5: Additional simulations to assess the impact of varying the effect of the IV on the exposure for the fractional polynomial method.

Simulation	Model	β	Fitting correct FP		Power of test		
			Mean (SD) [Mean SE]	Coverage	Quad	Q	FP
Low frequency	Linear	0	-0.001 (0.056) [0.057]	0.958	0.014	0.046	0.042
	Linear	1	0.999 (0.059) [0.058]	0.940	0.036	0.054	0.054
	Logarithm	2	1.811 (0.197) [0.183]	0.800	0.524	0.504	0.266
	Square root	2	1.894 (0.208) [0.207]	0.904	0.180	0.182	0.094
	Quadratic	0.1	0.110 (0.008) [0.008]	0.770	0.686	0.748	0.432
Super-additive	Linear	0	0.011 (0.077) [0.072]	0.922	0.014	0.056	0.072
	Linear	1	1.000 (0.074) [0.075]	0.952	0.040	0.058	0.064
	Logarithm	2	1.994 (0.238) [0.242]	0.946	0.472	0.446	0.224
	Square root	2	1.983 (0.279) [0.268]	0.950	0.144	0.144	0.076
	Quadratic	0.1	0.101 (0.010) [0.010]	0.938	0.524	0.532	0.270
Sub-additive	Linear	0	0.012 (0.095) [0.088]	0.924	0.014	0.052	0.062
	Linear	1	0.995 (0.089) [0.091]	0.958	0.044	0.056	0.068
	Logarithm	2	1.986 (0.279) [0.286]	0.952	0.360	0.318	0.170
	Square root	2	1.972 (0.336) [0.322]	0.948	0.112	0.108	0.070
	Quadratic	0.1	0.100 (0.012) [0.013]	0.946	0.372	0.380	0.186

Low frequency refers to the simulation setting where the IV is a low frequency variant (minor allele frequency = 0.03) with a large effect on the exposure (0.75). Super-additive refers to the simulation setting where the first allele of the genetic variant increases the exposure by 0.1 units and the second allele by 0.3 units. Sub-additive refers to the simulation setting where where the first allele of the genetic variant increases the exposure by 0.3 units and the second allele by 0.1 units. β is the effect parameter. Coverage refers to the number of replications where the true β was contained within the corresponding 95%CI. SD, standard deviation; SE, standard error; Quad, quadratic test for assessing non-linearity; Q, Cochran-Q test; FP; fractional polynomial test; IV, instrumental variable; CI, confidence interval.

Table S6: Additional simulations to assess the impact of varying the effect of the IV on the exposure for the piecewise linear method assuming a threshold effect with $\beta = 0.5$.

(a) Overall results

	Power of test		
Varying	Quad	Q	FP
Low Frequency	0.844	0.892	0.754
Super-additive	0.636	0.698	0.552
Sub-additive	0.406	0.536	0.372

(b) Quantile results

		Decile of the IV-free exposure distribution									
Simulation	Parameter	1	2	3	4	5	6	7	8	9	10
Low frequency	Correct	0.000	0.000	0.000	0.000	0.000	0.000	0.005	0.174	0.436	1.130
	Mean	0.005	0.010	0.012	0.017	0.036	0.085	0.184	0.348	0.615	1.313
	Coverage	0.948	0.946	0.942	0.938	0.928	0.852	0.678	0.742	0.786	0.886
Super-additive	Correct	0.000	0.000	0.000	0.000	0.000	0.000	0.028	0.198	0.459	1.153
	Mean	0.031	0.053	0.064	0.064	0.057	0.061	0.119	0.272	0.542	1.242
	Coverage	0.940	0.938	0.944	0.936	0.942	0.952	0.914	0.940	0.942	0.946
Sub-additive	Correct	0.000	0.000	0.000	0.000	0.000	0.000	0.049	0.219	0.480	1.174
	Mean	0.030	0.049	0.053	0.047	0.040	0.046	0.110	0.274	0.546	1.241
	Coverage	0.962	0.962	0.970	0.952	0.952	0.952	0.942	0.952	0.968	0.950

Low frequency refers to the simulation setting where the IV is a low frequency variant (minor allele frequency = 0.03) with a large effect on the exposure (0.75). Super-additive refers to the simulation setting where the first allele of the genetic variant increases the exposure by 0.1 units and the second allele by 0.3 units. Sub-additive refers to the simulation setting where where the first allele of the genetic variant increases the exposure by 0.3 units and the second allele by 0.1 units. Coverage refers to the number of replications where the true mean value of the outcome for that decile was contained within the corresponding 95% prediction interval. Quad, quadratic test for assessing non-linearity; Q, Cochran-Q test; FP; fractional polynomial test; IV, instrumental variable.
